# Misrepresentation about vaccines that are scaring women

**DOI:** 10.4102/phcfm.v13i1.2953

**Published:** 2021-06-09

**Authors:** Jagidesa Moodley, Olive P. Khaliq, Princess Z. Mkhize

**Affiliations:** 1 Department of Obstetrics and Gynaecology, Faculty of Clinical Medicine, University of KwaZulu-Natal, Durban, South Africa

**Keywords:** misrepresentation, vaccines, infertility, Social media, COVID-19

## Abstract

The coronavirus disease 2019 (COVID-19) pandemic has contributed greatly to morbidity and mortality worldwide. The production of COVID-19 vaccines has been tested for efficacy and safety via clinical trials. However, false information on the side effects of the vaccine has been spread via social media, creating fear of vaccination. Currently, the vaccine has been falsely reported to cause infertility in women of reproductive age and miscarriages in pregnant women. There is no evidence to support this information as the COVID-19 vaccines have been clinically approved for safety. Furthermore, pregnant and lactating women were not included in the clinical trials. Therefore, the objective of this report is to raise awareness that the rumours on the vaccine are false and to encourage every individual to accept the vaccination for their safety and the safety of their loved ones.

## Background

The coronavirus disease 2019 (COVID-19) pandemic has taken a disproportionate toll on women’s careers, finances and family lives. Because of the lockdown, many employees lost their jobs and some found it difficult to seek new jobs. Women are more likely to lose their jobs than men and are therefore less likely to get new jobs. Furthermore, women are less likely to be promoted than men.^1,2^ Vaccines may represent a solution in overcoming this pandemic but there are misrepresentations on social media that vaccination might have an impact on fertility or nursing of babies.^1^ This is of obvious concern as young women may decline vaccination or may omit the second dose. Evidence confirms that being vaccinated is the best way for women to protect themselves and their families.^1,2^ The confusion created by the misrepresentations is that as the clinical trials did not include pregnant and lactating women, safety data for these populations are not available.^2^

## Experience

Whilst we await results of the current COVID-19 vaccine trials that include pregnant and lactating women, we need to support women by providing scientific information, proving the benefit of COVID-19 vaccines. One study claims that the vaccines cause infertility by generating antibodies that not only target the coronavirus spike protein, as designed, but inadvertently react with a protein in the placenta called syncytin-1.^3^ It was reported that the viral protein and the human protein are so similar in structure that the protective antibodies against the coronavirus will prevent the placenta from developing properly, causing pregnancy loss.^4^ However, information stating that the coronavirus protein prevents placental development is incorrect, because there is no evidence that the coronavirus’ spike protein amino acid sequence is similar to that of the placental syncytin-1. There is also no evidence of reports of infertility amongst women who have recovered from COVID-19.^5^ On the contrary, women included in vaccine trials (vaccinated women) have unintentionally conceived whilst participating in the trials; therefore, this is a clear indication that the COVID-19 vaccine does not cause infertility.

Whilst there is little data and public messaging on coronavirus immunisation during breastfeeding, women are left to draw their own conclusion and some are assuming the worst. However, immunisation of lactating women benefits both the mother and the baby.^6^ Vaccines induce protective antibodies in the mother, which are passed onto babies via breast milk to help to protect them.^6^ It is unlikely that the vaccine can cross into breast milk. Even if it did, it would not pose a threat to the health of the baby, as the components would be degraded or digested in the stomach.^6^

Any immune response during pregnancy, whether from infection or a vaccine, could have unknown consequences. However, studies of COVID-19 vaccination in animals have shown no impact on embryogenesis and pregnancy as a whole.^7^ In addition, vaccines for seasonal influenza, tetanus and pertussis are routinely offered and safely administered to pregnant women. More recently, Fauci (2021) states that 20 000 pregnant women have been vaccinated in the United States (US) and none of these women have raised any red flags.^8^

Whilst we do not know everything about COVID-19 in human pregnancy, there is information that some women who contract the disease are at higher risk of pregnancy complications such as stillbirths, preterm labour and even death in the postpartum period.^9,10^ Although women are not more likely to be infected with COVID-19, pregnant women who become infected are more likely to develop adverse events than non-pregnant women.^9,10^ On the other hand, the chances of contracting COVID-19 from the vaccination are practically zero.^7,11,12^

Unfortunately, women are accepting misrepresentations because of the power of social media, from where they get information, much more quickly than getting answers to their questions from scientists.^2^ All pregnant women must be counselled by their doctors and provided with full information on COVID-19 infection and the role of COVID-19 vaccinations. Whatever choice women make should be supported by their health professional. Therefore, the scientific community needs to show that it is listening to and addressing public concerns.^13^

## Conclusion

The Centers for Disease Control (CDC) states that women will and should make their own decisions, but they should do this in an informed way.^14^ They cannot do so in an informed way without knowing the true risks and benefits of vaccination. For any woman who is pregnant, nursing or planning to conceive, contracting COVID-19 is certainly more dangerous than being vaccinated. Mass vaccination, combined with physical distancing and wearing of masks provides the only way to protect women, men and children from the disease.
